# Dealing with predatory journal articles captured in systematic reviews

**DOI:** 10.1186/s13643-021-01733-2

**Published:** 2021-06-11

**Authors:** Danielle B. Rice, Becky Skidmore, Kelly D. Cobey

**Affiliations:** 1 Department of Psychology, 2001 Avenue McGill College, McGill University, Montréal, Québec, Canada; 2 Centre for Journalology, Clinical Epidemiology Program, Ottawa Hospital Research Institute, 501 Smyth Road, Ottawa, Ontario Canada; 3 Center for Practice Changing Research, Ottawa Hospital Research Institute, The Ottawa Hospital, General Campus, 501 Smyth Road, Box 201b, Ottawa, Ontario K1H 8L6 Canada; 4 School of Epidemiology and Public Health, Faculty of Medicine, University of Ottawa, 75 Laurier Ave, Ottawa, Canada

**Keywords:** Predatory journals, Systematic reviews, Meta-analysis, Open access

## Abstract

**Background:**

Systematic reviews appraise and synthesize the results from a body of literature. In healthcare, systematic reviews are also used to develop clinical practice guidelines. An increasingly common concern among systematic reviews is that they may unknowingly capture studies published in “predatory” journals and that these studies will be included in summary estimates and impact results, guidelines, and ultimately, clinical care.

**Findings:**

There is currently no agreed-upon guidance that exists for how best to manage articles from predatory journals that meet the inclusion criteria for a systematic review. We describe a set of actions that authors of systematic reviews can consider when handling articles published in predatory journals: (1) detail methods for addressing predatory journal articles a priori in a study protocol, (2) determine whether included studies are published in open access journals and if they are listed in the directory of open access journals, and (3) conduct a sensitivity analysis with predatory papers excluded from the synthesis.

**Conclusion:**

Encountering eligible articles published in presumed predatory journals when conducting a review is an increasingly common threat. Developing appropriate methods to account for eligible research published in predatory journals is needed to decrease the potential negative impact of predatory journals on healthcare.

Systematic reviews are intended to critically appraise and synthesize the results of entire bodies of literature. In healthcare, they may be used in the development of clinical practice guidelines. The utility of systematic reviews, however, depends on the quality of the included studies [1]. An emerging methods concern among systematic review teams is that they may unknowingly capture studies published in “predatory” journals. This may degrade the quality of a systematic review, and in medicine, has the potential to create downstream effects that negatively impact patient care.

To the unacquainted, predatory journals are “entities that prioritize self-interest at the expense of scholarship and are characterized by false or misleading information, deviation from best editorial and publication practices, a lack of transparency, and/or the use of aggressive and indiscriminate solicitation practices” [2]. Predatory journals often exploit the open access (OA) publishing model, where copyrights are typically retained by authors, and work is free to access and build upon [3]. OA publishing commonly requires authors to pay an article processing charge (APC) if their paper is accepted for publication [4]. Predatory journals may promote themselves as “OA” to earn a profit from APCs, but without meeting expected publication best practices. For example, they may not provide peer review [5], meaning work published is not vetted. Peer review can improve the quality and usability of research findings. If a predatory journal states it conducts peer review, but it is not occurring, this has the potential to create bias. Examples of the public making medical decisions based on articles published in predatory journals have been noted previously [2, 6]. Further, a study examining 1907 biomedical studies in presumed predatory journals demonstrated that less than half of the included studies reported having ethics approval, yet more than two million humans were included in the predatory publications [7].

Research published in predatory journals is often difficult to retrieve through database searches as these journals are indexed less commonly than traditional journals, suggesting that when legitimate quality research gets published in predatory journals, patient participation in research, use of animals in research, and funds used for the research including paying APCs, may be wasteful. The quality of articles published in predatory journals has been studied in a sample of 1907 biomedical studies in presumed predatory journals [7]. Overall, the quality of articles was far worse than research published in non-predatory journals. Within this sample, however, there were articles that were of similar quality to research published in non-predatory journals. For example, 14% of trials (*n* = 13) were registered. Being unable to retrieve these articles due to a lack of indexing in common databases, or choosing to disregard these trials solely due to being published in a predatory journal may impact the conclusions drawn during a systematic review and does not fulfill the goal of a systematic review in providing a comprehensive overview of all eligible studies. The counterpoint to this is that availability of findings in predatory journals also poses a threat, since this could result in unvetted research being used, and in the case of systematic reviews, potentially then integrated into clinical decision making. A recent study reported that articles in suspected predatory journals received fewer citations than those in legitimate journals. The authors concluded that the average predatory journal article has very little effect on the research of others and has limited readership [8]. We disagree with their conclusion since (1) failure to retrieve quality work published in a predatory journal contributes to location bias and publications bias of which detrimental effects are well documented [9–11] and (2) retrieving poorly conducted research published in presumed predatory outlets that have not been vetted for accuracy, quality, or ethical approval poses harms to patients if applied by clinicians intending to use evidence-informed decision making [2]. Results from articles published in predatory journals have been included in systematic reviews previously and can alter findings and recommendations made based on knowledge synthesis [12, 13]. The presence of predatory journals in databases has been increasing in recent years with Google Scholar and PubMed, one of the world’s leading biomedical databases, both including articles from predatory journals [12, 14].

Two of us (DBR and BS) recently conducted a systematic review and network meta-analysis aimed to compare the effectiveness of psychosocial therapies among people receiving opioid agonist treatment [15]. The search (see https://osf.io/w9azr/?view_only=0658ab3734414562a6f0eeeee506371f) for this review was developed by an experienced medical information specialist (BS) in consultation with the review team. The MEDLINE strategy was peer reviewed using the PRESS guideline. The search did not have any language restrictions; however, eligible articles had to be published in English or French. A total of 71 RCTs were eligible for inclusion in the review. When piloting the risk of bias assessments, two reviewers noticed one trial that stood out as being particularly poorly reported [16]. This study had a high risk of bias and included results that were discrepant between the text and tables, as well as grammar and spelling errors.

We reviewed the website of the journal that published the low-quality trial [16] and the journal was presumed to be predatory. Based on our concerns with this trial [16], we decided to review all included trials to determine if journals were subscription-based, hybrid (with subscriptions and the option for open access publishing), or exclusively open access. For any OA journals, we checked to see if the journal was listed in the Directory of Open Access Journals (DOAJ) and noted its presence or absence. Eight of the 71 included trials were published in OA journals; four (50%) of these OA journals were not listed in the DOAJ and had more than 1 year’s worth of research content. These journal websites also contained spelling errors and atypical statements such as the journal being approved by the minister of health. The articles were published in 2013, 2017, 2017, and 2018 and each of the four studies had a high risk of bias. These trials published in potentially predatory journals were retained in our review to capture all relevant evidence and reduce the risk of publication bias in our review. However, a sensitivity analysis that excluded these studies was then conducted and no differences were found as compared to the primary analysis.

We suspect our experience encountering presumed predatory journals during the conduct of a systematic review is increasingly common. This begs the question: what actions should systematic review teams take to deal with presumed predatory journal articles? We know of no applicable formal guidance. In Table 1, we list some suggested actions. To fulfill the goals of a systematic review by critically appraising and synthesizing results from an entire body of literature, the inclusion of articles published in predatory journals is necessary. Systematic reviews should apply standardized approaches to identify articles that are eligible for a systematic review, including those published in predatory journals and conduct thorough risk of bias assessments to determine the impact of these articles in the synthesis of findings. Facilitating sensitivity analyses and considering results both with and without research from predatory journals may be an important step for future systematic reviews. Failure to search for and include articles published in potentially predatory journals contributes to research waste, publication bias, and incomplete evidence synthesis. Suggested approaches are noted during the protocol and review stage for systematically managing and including eligible articles (see Table 1). During the review, the focus of suggested actions center on assessing the likelihood that an article is published in a predatory journal. The suggested approach involves checking multiple websites to determine if an article (1) is published in an OA journal, (2) is listed in the DOAJ, and (3) is a member of COPE (Committee On Publication Ethics). These suggested steps can be completed at the time of data extraction to decrease additional workload. These recommendations allow articles in potentially predatory journals to be included in systematic reviews while taking into consideration the poor quality that is common among articles published in potentially predatory journals.

**Table 1:** Suggestions for how systematic reviewers can deal with predatory journals

For the review protocol: 1. Detail your methods for addressing the potential for predatory journal articles being captured in your search. (a) Specify how you will determine if an included article meets the criteria for being in a “predatory” journal [17]. (b) Note how you will deal with included articles you determine to be from “predatory” journals. For the review: 1. Determine whether included studies are published in open access journals. To do so we suggest the following: (a) If included studies are published in open access (OA) journals, check to determine if the journal is listed in the DOAJ. If yes, presume the journal is legitimate. (b) If included OA journals are not listed in the DOAJ, check to see if the journal is a member of COPE (Committee On Publication Ethics). Note that you should check the COPE membership directory, rather than assume a statement of membership on a journals website is accurate. If yes, presume the journal is legitimate. (c) If included OA journals are not in the DOAJ and not COPE members, review the journal website for characteristics of predatory journals [2]. We suggest that if two or more salient features of predatory journals are present that the journal be classified as predatory. 2. For quantitative analyses, conduct a sensitivity analysis with predatory papers excluded from the synthesis. 3. For qualitative analyses, synthesize results both with and without predatory papers included. 4. Discuss the presence and implications of predatory papers, where relevant.	

Planning to search for, address, and include articles in potentially predatory journals prior to beginning a systematic review is important to minimize bias. The preferred reporting items for systematic review and meta-analysis protocols (PRISMA-P) is a checklist intended to facilitate the preparation and reporting of a robust protocol for systematic reviews. PRISMA-P includes items related to search strategy, data collection, and risk of bias [18]. Updates of PRISMA-P may consider incorporating data collection for the type of journals included in systematic reviews to systematically assess the presence of potentially predatory journals in future systematic reviews and meta-analyses. Assuring that appropriate methods are in place to account for eligible research published in predatory journals while conducting systematic reviews is necessary to decrease the potential negative impact of predatory journals on healthcare. Future research that considers the best approach to manage the inclusion of articles published in potentially predatory journals will allow for increased consistency across systematic reviews.

## Data Availability

Data sharing is not applicable to this article as no datasets were generated or analyzed during the current study.

## Abbreviations

DOAJ: Directory of Open Access Journals
OA: Open access
PRISMA-P: Preferred reporting items for systematic review and meta-analysis protocols
APC: Article processing charge
PRESS: Peer review of electronic search strategies
